# Epiregulin promotes hair growth via EGFR‐medicated epidermal and ErbB4‐mediated dermal stimulation

**DOI:** 10.1111/cpr.12881

**Published:** 2020-07-22

**Authors:** Nahyun Choi, Won‐Serk Kim, Sang Ho Oh, Jong‐Hyuk Sung

**Affiliations:** ^1^ STEMORE Co. Ltd. Incheon South Korea; ^2^ College of Pharmacy Yonsei Institute of Pharmaceutical Sciences Yonsei University Incheon Korea; ^3^ Department of Dermatology Kangbuk Samsung Hospital Sungkyunkwan University School of Medicine Seoul South Korea; ^4^ Department of Dermatology Severance Hospital and Cutaneous Biology Research Institute Yonsei University College of Medicine Seoul South Korea

## Abstract

**Objectives:**

EREG (epiregulin), a member of the epidermal growth factor (EGF) family, plays a role in inflammation, wound healing, normal physiology and malignancies. However, little is known about its function on hair growth.

**Materials and Methods:**

Cell growth assay, QPCR and immunostaining were carried out. Telogen‐to‐anagen transition and organ culture were conducted. ROS level was monitored by staining DCFDA.

**Results:**

We investigated the hair inductive effect of EREG and the mechanism of stimulation on DPCs and ORS cells during hair cycling. Whereas EREG promoted hair growth, EREG knockdown inhibited hair growth as evidenced by telogen‐to‐anagen transition and organ culture models. EREG was expressed in epidermal cells including ORS cells in vivo. EREG activated phospho‐ErbB4 in DPCs during hair cycling and stimulated DPCs via ErbB4 activation in vitro. In terms of the underlying mechanism, reactive oxygen species (ROS) played a key role in DPC stimulation. EREG also activated phospho‐EGF receptor (EGFR) in epidermal cells including matrix and ORS cells in vivo and stimulated ORS cells via EGFR activation in vitro.

**Conclusions:**

EREG, which is released from ORS cells, activated EGFR and ErbB4 on epidermal cells and DPCs during hair cycling, respectively. As a result, EREG stimulated epidermal cells a positive feedback and DPCs via regulating ROS generation for hair growth. Therefore, EREG therapy may be a novel solution for hair loss treatment.

## Graphical abstract

EREG was mainly expressed and released from epidermal cells including ORS cell at anagen stage. EREG‐EGFR activation stimulated matrix and ORS cells for hair growth. EREG‐ErbB4 activation also stimulated DPCs through the regulation of NOX4‐ROS level, thereby stimulating hair growth.

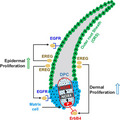

## 1 INTRODUCTION

Hair loss is a disease caused by aged hair follicles as a result of internal factors such as ageing and hormones, and external factors such as stress and environmental hormones. Once hair loss begins, it is progressive and irreversible, and there is currently no fundamental treatment for hair loss. The treatment options used so far include stimulating existing hair follicles so that new hair grows faster and richer, or transplanting new hair. Another option is to use cell therapy. It has been reported that adipose‐derived stem cells (ASCs) and their conditioned medium (CM) have hair growth effect, but results have been largely unsatisfactory until now. Therefore, previous studies have focused on enhancing the function of ASCs to promote hair growth.^1^, ^2^, ^3^, ^4^, ^5^, ^6^ In particular, the biggest reason why ASCs and their CM can increase hair growth is because many growth factors are released from ASCs to promote hair growth. We recently studied the enhancement of ASC function by heparin‐binding epidermal growth factor (EGF)‐like growth factor (HB‐EGF), which is one of the EGF family growth factors, and its hair growth effect.^6^ Among the other members of the EGF family, EREG has also stimulated ASC motility, and EREG‐pre‐conditioned ASC and its CM enhanced hair growth (data not shown). When we examined whether HB‐EGF and EREG themselves had hair growth effect, only the subcutaneous injection of EREG induced hair growth. It was expected that EREG would probably act directly on the cells that make up the hair follicles to increase hair growth.

The mature growth factors bind to members of the EGF receptor (ErbB) family of receptor tyrosine kinases (RTKs). This family consists of EGFR, ErbB2, ErbB3 and ErbB4. Among them, EREG has a high binding affinity for EGFR and ErbB4.^7^ A binding of ligand with ErbB receptors can either homodimerize or heterodimerize with a different member of the ErbB family. Receptor dimerization enables the tyrosine phosphorylation of one receptor by the other. This cross‐phosphorylation creates binding sites for effector proteins, thereby enabling the receptors to serve numerous signalling cascades, most notably the Raf/Ras/mitogen‐activated protein kinase signalling (MAPK) and the phosphoinositide 3 kinase (PI3K)/Akt pathway.^8^, ^9^ The network of EGF family and ErbB receptors regulates the proliferation, differentiation, inflammation, wound healing and function of numerous tissues in humans, and deregulated signalling of this network is a hallmark of several different human malignancies.^10^, ^11^ Recently, it was reported that human EREG (hEREG) treatment induced hair shaft elongation regulating keratinocyte proliferation.^12^ However, investigation of the mechanisms of how EREG induces hair growth is still required. Therefore, the aim of this study is to investigate the function of EREG and the associated mechanism during hair cycling.

## 2 MATERIALS AND METHODS

### 2.1 Cell culture

Human dermal papilla cells (DPCs), purchased from PromoCell (#C‐12071), were cultured in follicle DPC medium with supplement mix (PromoCell) and 0.1% anti‐antibiotics (Gibco). After informed consent was obtained, human outer root sheath cells (ORSs) were isolated (Yonsei University College of Medicine, 4‐2018‐0141). Human hair follicle was extracted, and the ORS part was cultured for 2‐3 days in a culture dish containing DPC medium with supplement mix. Next, proliferating cells were subcultured with ORS medium. The ORS was cultured in EpiLife medium (Gibco) with EpiLife™ Defined Growth Supplement (EDGS; Gibco) and 1% penicillin/streptomycin (Gibco). DPCs and ORSs for all experiments were used at passages 3‐4 and 1‐2, respectively. All cells were maintained at 37°C in a humidified 5% CO_2_ incubator.

### 2.2 Mouse telogen‐to‐anagen induction

The mice were maintained and anesthetized according to a protocol approved by the US Pharmacopoeia and the Institutional Animal Care and Use Committee of Yonsei University (IACUC‐201712‐681‐02). The dorsal region of 7‐week‐old male C3H/HeN mice in the telogen stage of the hair cycle was shaved and depilated using an electric shaver, with special care taken to avoid damaging the bare skin. Then, 100 ng/mL of EREG (PeproTech) was injected daily into the dorsal skin of the shaved mice. Negative control or siRNA for EREG (Bioneer; 480 pmol/1 mice) was injected with in vivo‐JetRNA (Polyplus Transfection, Inc) into the dorsal skin of shaved mice for a total of four times. For EREG‐treated DPC injection experiment, 4 × 10^4^ of the control DPC or preconditioned DPC with EREG (20 ng/mL) for 4 days were injected into the dorsal skin of the shaved mice. After the mice was first shaved, the actual length of the shaved part was measured. Almost all mice were shaved with size of approximately 2 × 4.3 cm, and only the weight of new hair in the shaved area was measured after sacrifice. Any darkening of the skin (indicative of hair cycle induction) was carefully monitored using image capture. After approximately 16‐18 days, the dorsal hair was shaved and weighed to estimate growth rate, and the dorsal skin was analysed using HE staining and immunostaining.

## 3 RESULTS

### 3.1 EREG induces hair growth in vivo

**Figure 1:**
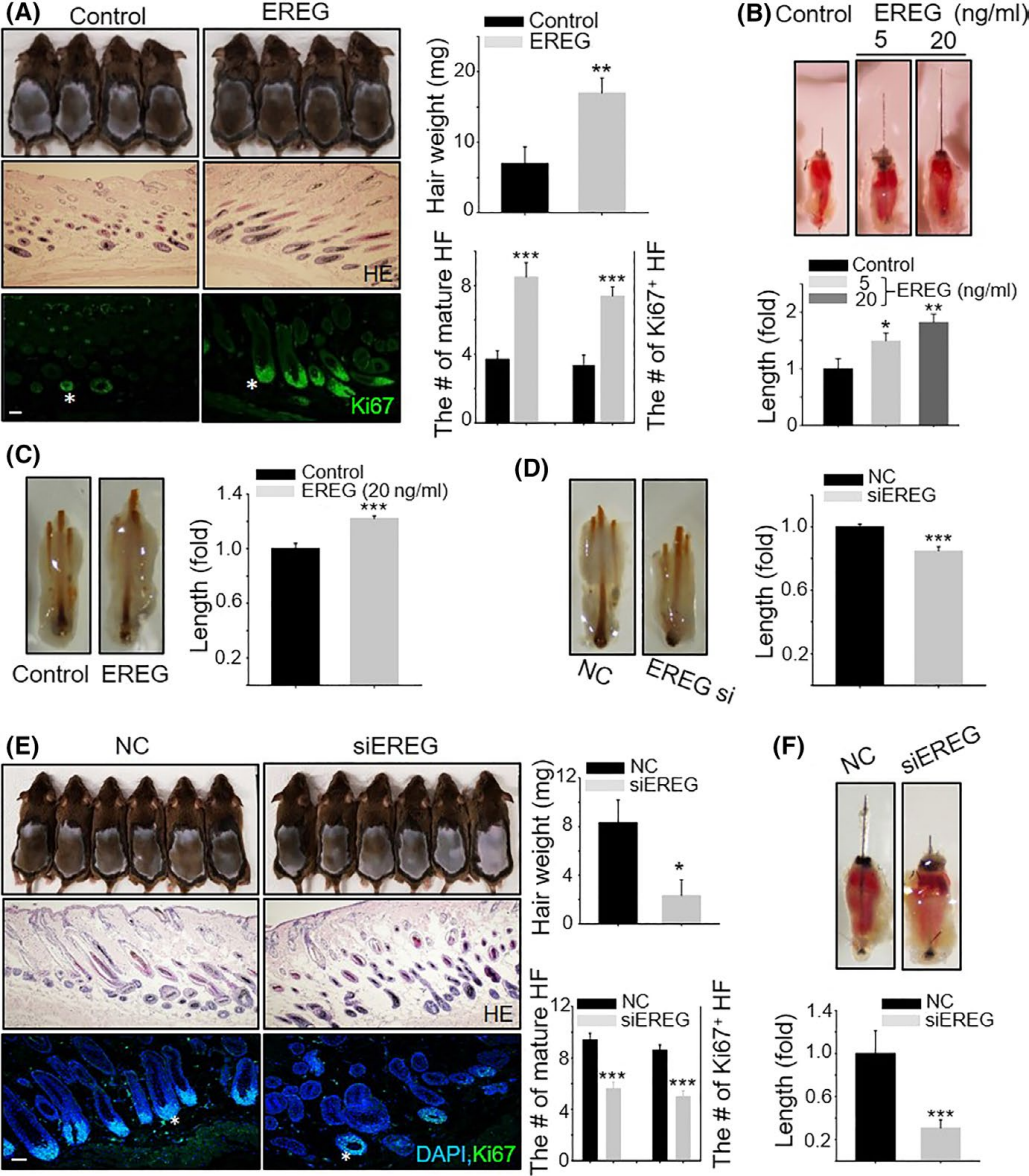
EREG promotes hair growth in vivo and ex vivo. A, Human recombinant EREG (100 ng) was injected into the dorsal skin of shaved 7‐wk‐old mice. Images were captured, and hair weight was measured 14 d later. Skin section was analysed using haematoxylin and eosin (HE) staining, and the number of mature hair follicles (HF) was measured. Hair follicle with Ki67^+^ matrix cells was shown by immunostaining. Asterisks indicate hair follicles with Ki67^+^ matrix cells (n = 4 per group). All error bars indicate the standard error of the mean (SEM); B, EREG (5 and 20 ng) increased the length of mouse vibrissal hair follicles compared with the control ex vivo; C, EREG (20 ng) increased the length of pig skin hair follicles compared with the control ex vivo; D, F, EREG siRNA reduced the length of mouse vibrissal and pig skin hair follicles compared with the negative control ex vivo; E, EREG siRNA was injected into the dorsal skin of shaved 7‐wk‐old mice. Images were captured, and hair weight was measured 16 d later. Skin section was analysed using HE staining, and the number of mature HF, hair follicle with Ki67^+^ matrix cells, was shown by immunostaining. Asterisks indicate hair follicles with Ki67^+^ cells in the cortex regions. *P < .05, **P < .01, ***P < .001. Three independent experiments were conducted for all data points. All scale bars indicate 10 μm

In contrast to HB‐EGF‐preconditioned ASCs, which promote hair growth in vivo, thereby stimulating ASC motility,^6^ direct injection of HB‐EGF itself did not have a hair growth effect (Figure S1). However, the subcutaneous injection of EREG promoted telogen‐to‐anagen induction (Figure 1A) and the length of mouse vibrissa and pig skin hair follicles (Figure 1B,C and Figure S2). To further confirm the hair promotion effect of EREG, we investigated whether EREG knockdown would have an opposite effect on hair growth. The reduced expression of EREG in EREG siRNA‐injected mice was detected in the epidermis and ORS cells compared to that of negative control mice (Figure S3). Indeed, EREG knockdown induced an opposite effect on hair growth as evidenced by telogen‐to‐anagen transition (Figure 1E) and organ cultures (Figure 1D,F and Figure S2). These results suggested that EREG is capable of promoting hair growth in vivo.

### 3.2 EREG was expressed in epidermal cells including ORS cells during hair cycling in vivo

**Figure 2:**
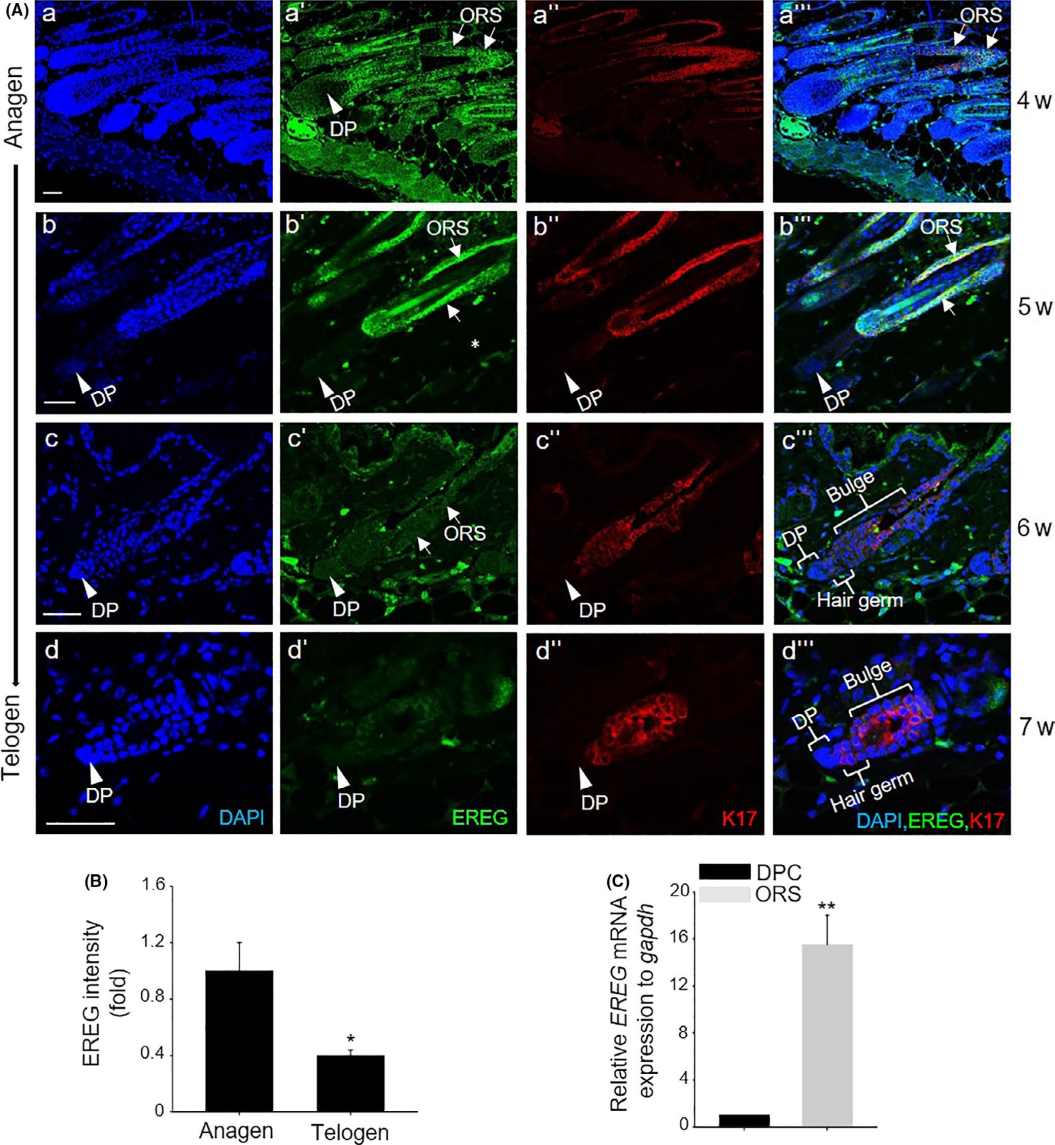
EREG was expressed in ORS cells. A, The strong EREG expression (green) was observed in ORS cells (arrow), which is co‐stained with cytokeratin17 (K17, red) at anagen stage. On the contrary, a very weak EREG expression was also observed in ORS cells at telogen stage (c–d'''). A very faint EREG expression was detected in hair germ and bulge cells, which will later become ORS cells (d–d'''). Any EREG expression was not detected in DPC (arrow head); B, the intensity of EREG expression was monitored during hair cycling using Image J program; and C, the expression level of EREG was compared in dermal papilla cells (DPCs) and ORS cells. *P < .05, **P < .01. Three independent experiments were conducted for all data points. All scale bars indicate 10 μm

Next, we investigated the expression pattern of EREG during hair cycling. To do this, we examined EREG expression during anagen, catagen and telogen (4‐7 weeks) stages. EREG was expressed strongly in epidermal cells including ORS cells (Figure 2A), and this expression was co‐stained with keratin17, a marker for ORS cells, at the anagen stage (Figure 2A, a‐a‴). The expression of EREG was not detected in the DP region at anagen and early catagen (Figure 2A, a‐b'''). The expression of EREG is obviously higher during the anagen stage and lower during the telogen stage; this is also confirmed by monitoring the expression intensity during hair cycle (Figure 2A,B). The very weak EREG expression was detected in hair germ cells and bulge region cells, which are the cells that will later differentiate ORS, at telogen stage, not in the DP region (Figure 2A, d‐d'''). The strong EREG expression in ORS cells was also confirmed by checking the mRNA expression level between DPCs and ORS cells in vitro (Figure 2C). These results suggested that EREG is mainly expressed and secreted from epidermal cell including ORS cells.

### 3.3 Activation of EREG receptor stimulated ORS cells and DPCs differentially in vivo

**Figure 3:**
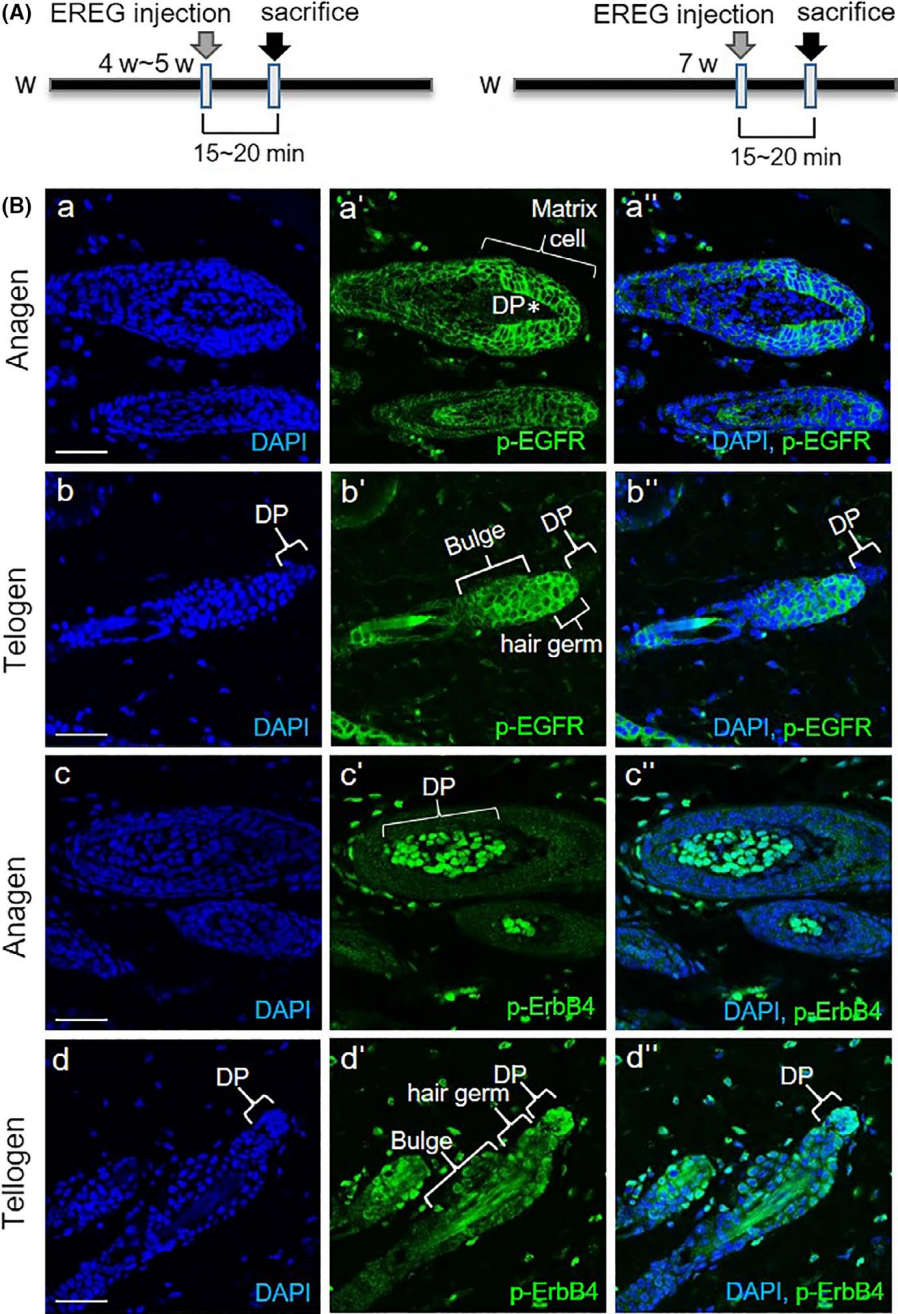
Differential activation of EREG receptors on keratinocytes including matrix and ORS cells, and DPCs. A, Schematic diagram for monitoring of receptor phosphorylation after EREG subcutaneous (sc) injection. Phosphorylation of EGFR1 or ErbB4 was monitored at 15‐20 min after EREG injection into the dorsal skin of shaved mice; B, p‐EGFR expression was observed in keratinocytes including matrix and ORS cells at anagen (a‐a''), and hair germ and bulge cells at telogen (b‐b''). No p‐EGFR‐positive cells were detected in DPCs. p‐ErbB4 expression is shown in DPCs at anagen (c‐c''). Moreover, strong p‐ErbB4 expression in DPCs and weak expression in hair germ and bulge cells at telogen were detected (d‐d''). All scale bars indicate 10μm

Among the ErbB family of receptors, EREG has a high binding affinity to EGFR and ErbB4.^13^ To determine where the activation of EREG receptor occurred and which receptor binds to EREG in vivo, we investigated the expression of phospho‐EGFR or phospho‐ErbB4 after subcutaneous injection of EREG (Figure 3A). The expression of phospho‐EGFR was detected in ORS cells of entire hair follicles, and a stronger expression was detected in matrix cells 15‐20 min after EREG injection at the anagen stage (Figure 3B, a‐a''). The expression of phospho‐EGFR was also detected in hair germ cells and bulge region cells at telogen stage (Figure 3B, b‐b''). No phospho‐EGFR‐positive cell was detected in DP cells during anagen and telogen stages (Figure 3B, a‐b''). EREG binds to EGFR mainly on matrix cells and ORS cells at anagen, and hair germ cells and bulge region cells at telogen. These results suggested that EREG‐EGFR activation occurred on matrix cells and ORS cells at anagen, and hair germ cells and bulge cells at telogen.

Meanwhile, a strong expression of phospho‐ErbB4 was detected in DPCs 20 minutes after EREG injection, and not in matrix and ORS cells at anagen stage (Figure 3B, c‐c''). A strong expression of phospho‐ErbB4 was also detected in DPCs, and a relatively weak expression was observed in hair germ cells and bulge region cells at telogen (Figure 3B, d‐d''). These results suggested that EREG‐ErbB4 activation occurred mainly in DPCs during hair cycling. All these data suggested that secreted EREG from epidermal cells including ORS cells might bind to EGFR on matrix and ORS cells, and ErbB4 on DPCs may thus transduce the signal for hair growth.

### 3.4 Activation of EREG‐ErbB4 stimulated DPCs

**Figure 4:**
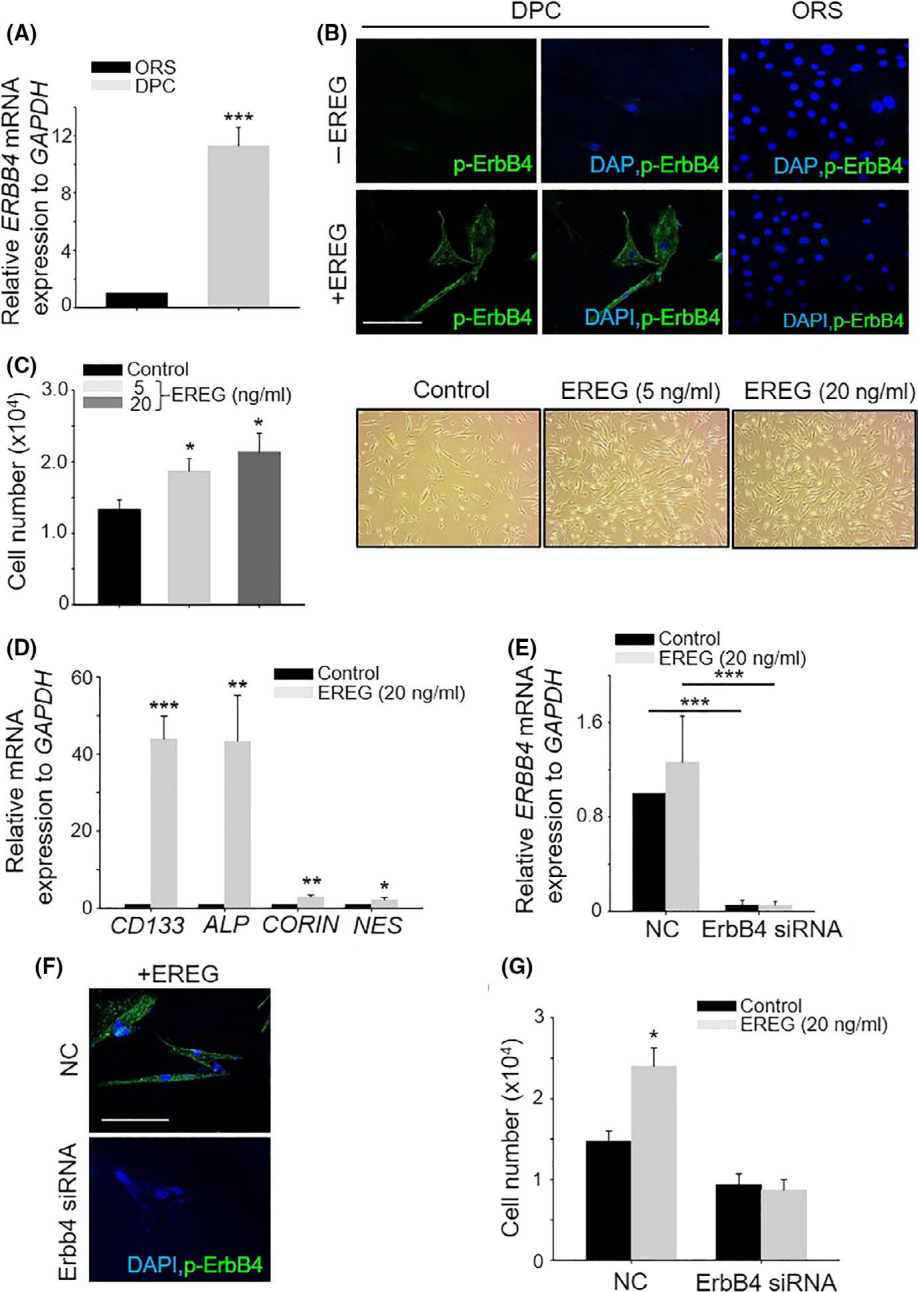
EREG stimulated DPCs by activation of ErbB4 receptor. A, mRNA level of ERBB4 was compared with DPCs and ORS cells in vitro; B, p‐ErbB4 expression was detected 15 min after EREG (20 ng/mL) treatment in more than 95% of DPCs analysed, whereas it was not detected in ORS cells after EREG treatment; C, cell growth after EREG treatment (5, 20 ng/mL) was monitored in DPCs; D, expression of DPC marker genes, *CD133*, *ALP*, *CORIN*, *NES*, was examined using qPCR after EREG treatment in DPCs; E, expression level of ErbB4 mRNA was checked between negative control for siRNA‐ and ErbB4 siRNA‐treated DPCs; F, p‐ErbB4 expression was checked between negative control and ErbB4 siRNA group after EREG treatment (20 ng/mL); and G, cell growth after EREG treatment was monitored in negative control‐ and ErbB4 siRNA‐treated DPCs. *P < .05, **P < .01, ***P < .001. Three independent experiments were conducted for all data points. All scale bars indicate 10 μm

As EREG phosphorylated ErbB4 on DPCs in vivo, we investigated whether EREG can stimulate DPCs via ErbB4 activation in vitro. First, the expression level of ERBB4 mRNA was significantly increased in DPCs compared with that in ORS cells (Figure 4A). In addition, more than 95% of phospho‐ErbB4 positive cells were detected in DPCs 15 minutes after EREG treatment (Figure 4B). However, no phospho‐ErbB4 positive cells were detected in ORS cells even after EREG treatment (Figure 4B). EREG also stimulated DPCs, as evidenced by the proliferation and expression level of DPC marker genes. EREG dose‐dependently increased DPC proliferation by up to 2‐fold (Figure 4C) and increased the mRNA expression level of DPC marker genes such as *CD133*, *ALP* (alkaline phosphatase), *CORIN* and *NES* (nestin) (Figure 4D). In particular, when ALP staining was performed to check the activity of ALP, the number of ALP‐positive cells was increased after EREG treatment (Figure S4). Moreover, EREG‐pre‐conditioned DPCs promoted hair growth effect significantly in vivo evidenced by hair weight, the number of hair follicle and hair follicle with Ki67^+^ matrix region (Figure S5) suggesting stimulated DPCs by EREG promoted hair growth. All of these results indicated that EREG stimulated DPCs thereby promoting hair growth.

To elucidate whether the stimulation of DPCs by EREG was caused by ErbB4 activation, we reduced the ErbB4 mRNA level using ErbB4 knockdown. The results confirmed that the expression level of ErbB4 mRNA was reduced after treatment with ErbB4 siRNA (Figure 4E). Whereas a strong phospo‐ErbB4 expression was observed in the negative control group after EREG treatment, no strong phospo‐ErbB4 expression was detected in the ErbB4 siRNA group despite the treatment with EREG (Figure 4F). Moreover, cell proliferation in ErbB4 knockdown DPCs was not increased by EREG treatment (Figure 4G). These results suggested that EREG phosphorylated ErbB4 receptor on DPCs, thereby stimulating DPCs.

### 3.5 EREG increased reactive oxygen species levels by regulating the expression of NADPH oxidase 4 in DPCs

**Figure 5:**
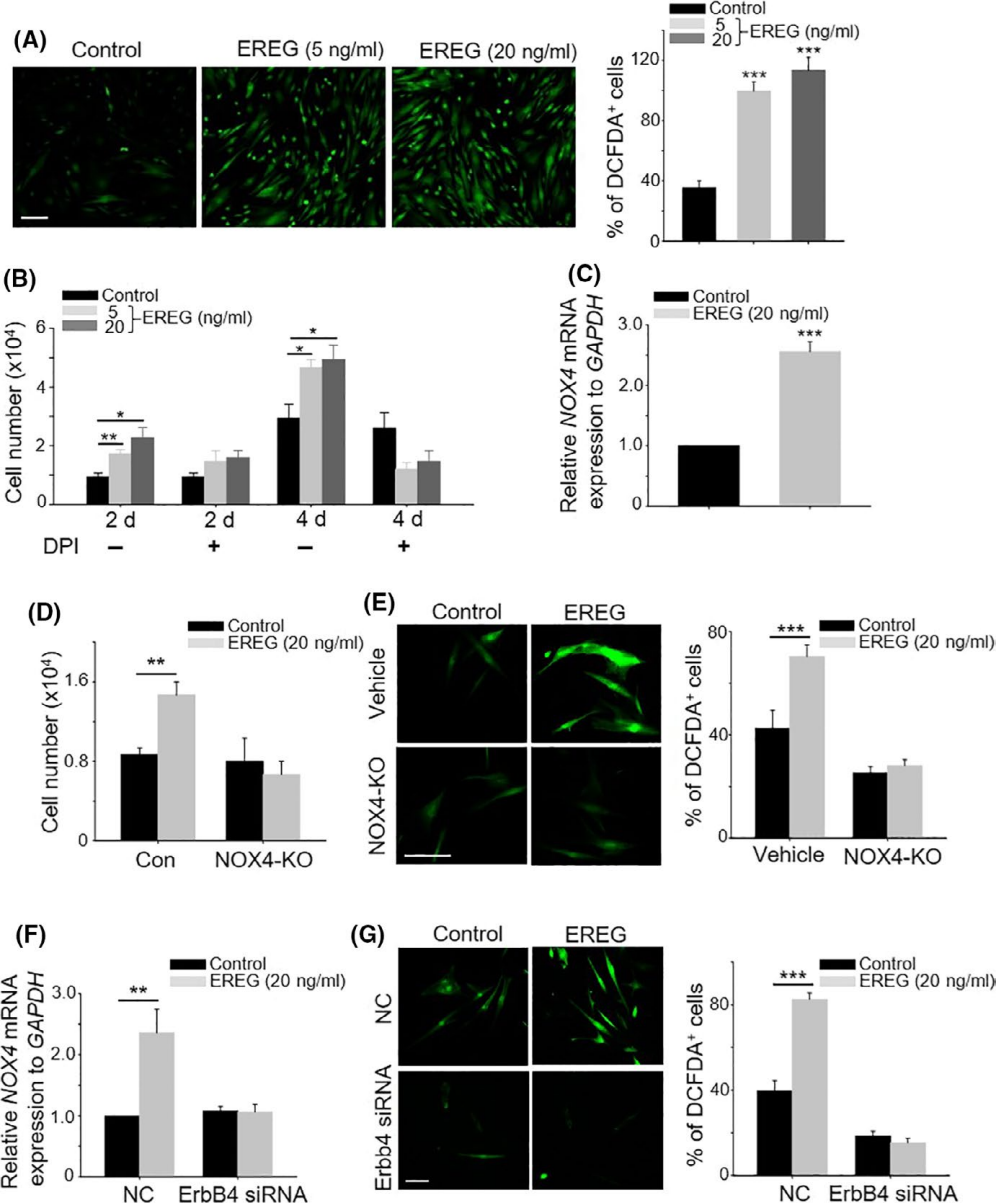
EREG increase ROS levels by regulating NOX4 expression in DPCs. A, Cellular ROS levels were monitored after EREG treatment using DCFDA staining; B, increased cell growth by EREG was monitored after DPI treatment; C, expression level of NOX4 mRNA was examined after EREG treatment using qPCR reaction; D, cell growth after EREG treatment was monitored in NOX4 knockout DPCs; E, ROS levels were monitored after EREG (20 ng/mL) treatment using DCFDA staining in NOX4 knockout DPCs; F, expression level of NOX4 mRNA was examined after EREG treatment in ErbB4 knockdown DPCs using qPCR; and G, ROS levels were monitored after EREG (20 ng/mL) treatment using DCFDA staining in ErbB4 knockdown DPCs. *P < .05, **P < .01, ***P < .001. Three independent experiments were conducted for all data points. All error bars indicate the SEM. All scale bars indicate 10 μm

Previous studies have shown that hypoxia improves hair inductivity of DPCs via nuclear NADPH oxidase 4 (NOX4)‐mediated reactive oxygen species (ROS) generation.^14^ Therefore, to investigate whether the stimulation of DPCs by EREG is associated with increased cellular ROS levels, we examined the cellular ROS levels after EREG treatment using 2′,7′‐dichlorofluorescin diacetate (DCFDA) staining. EREG treatment significantly increased the cellular ROS level (Figure 5A); however, this increase is not attributable to the increased ROS production in mitochondria at least (Figure S6). This is because when EREG‐treated DPCs were co‐stained with MitoTracker, which is a mitochondria marker, and MitoSOX, which is an ROS indicator in mitochondria, MitoSOX was not detected in mitochondria. Rather, some of the MitoSOX signals are detected inside the nucleus. These results are in line with previous reports about the increased NOX4 expression in the nucleus, and not in mitochondria of DPCs.^14^ To examine whether increased cellular ROS levels can regulate DPC proliferation by EREG, cell proliferation was measured after diphenyleneiodonium (DPI, a NADPH oxidase inhibitor) treatment. DPI treatment attenuated the increase of DPC proliferation by EREG (Figure 5B), suggesting that increase of DPC proliferation by EREG is attributable to NADPH oxidase level. To know which NADPH oxidase is related with the above result, the expression levels of all NOX families (NOX1‐NOX5) were checked after EREG treatment. NOX1, NOX2, NOX3 and NOX5 were not detected in both EREG‐treated and EREG‐untreated DPCs (Figure S7). In effect, only the expression level of NOX4 is regulated by EREG treatment (Figure 5C). These results are consistent with previous results indicating that NOX4 is a key regulator in increasing ROS levels among other NOX families.^14^ Next, the role of NOX4 in increased cell proliferation and ROS level by EREG was investigated using NOX4 knockout DPCs. Results showed that cell proliferation and ROS level in NOX4 knockout DPCs were not increased by EREG treatment, whereas EREG increased cell proliferation and ROS level in the control DPCs (Figure 5D,E).

Finally, to elucidate whether ErbB4 attenuation can affect the expression of NOX4 mRNA and ROS level by EREG, the expression level of NOX4 mRNA and ROS level were monitored in ErbB4 knockdown DPCs with or without EREG. The expression level of NOX4 mRNA was increased by EREG in the negative control group, whereas the expression level of NOX4 mRNA was not increased by EREG in ErbB4 siRNA (Figure 5F). The ROS level was significantly decreased in ErbB4 knockdown DPCs and was not increased by EREG treatment (Figure 5G). In contrast, EREG increased the ROS level in negative control DPCs (Figure 5G). These results indicate that activation of EREG‐ErbB4 increased the production of ROS by regulating NOX4 expression.

### 3.6 Activation of EREG‐EGFR stimulated ORS cells

**Figure 6:**
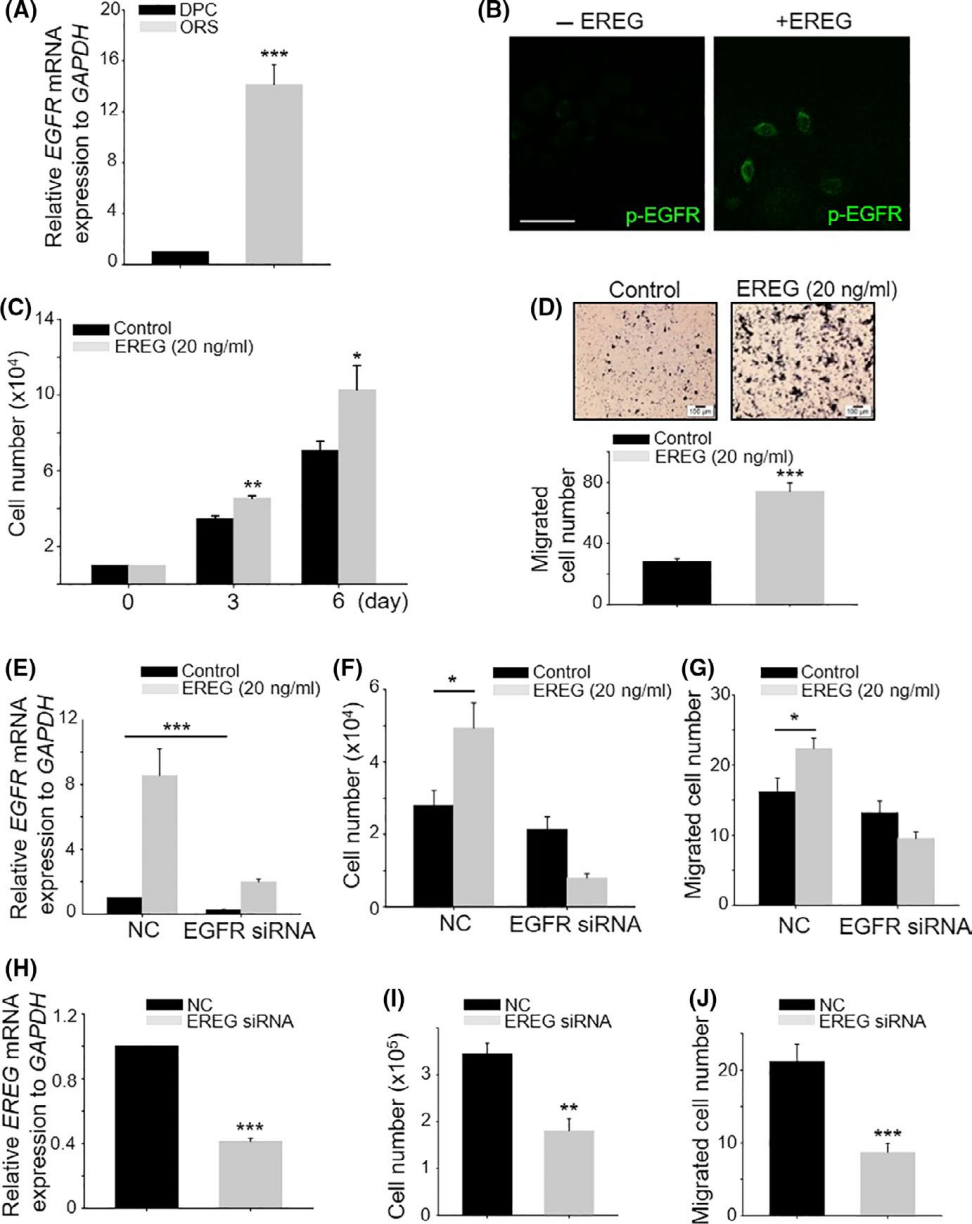
EREG stimulated ORS cells via activation of EGFR. A, mRNA level of EGFR was compared with DPCs and ORS cells in vitro; B, p‐EGFR expression was detected 15 min after EREG (20 ng/mL) treatment in DPCs. Scale bar indicates 10 μm; C and D, cell growth and migratory ability after EREG treatment (20 ng/mL) were monitored in ORS cells; E, expression level of EGFR mRNA was checked between negative control for siRNA‐ and EGFR siRNA‐treated ORS cells; F and G, cell growth and migratory ability after EREG treatment (20 ng/mL) were monitored in negative control and EGFR knockdown ORS cells; H, expression level of EREG mRNA was checked between negative control for siRNA‐ and EREG siRNA‐treated ORS cells; and I and J, cell growth and migratory ability were monitored in negative control and EGFR knockdown ORS cells. *P < .05, **P < .01, ***P < .001. Three independent experiments were conducted for all data points

As EREG phosphorylated EGFR in matrix and ORS cells in vivo (Figure 3B), we investigated whether EREG can stimulate ORS cells in vitro. First, the expression level of EGFR mRNA was significantly increased in ORS cells compared with that in DPCs (Figure 6A). Phospho‐EGFR expression was detected in ORS cells 15 minutes after EREG treatment (Figure 6B). EREG also stimulated ORS cells as evidenced by proliferation and migration (Figure 6C,D). These results indicated that EREG stimulated ORS cells.

To elucidate whether stimulation of ORS cells by EREG was caused by EGFR activation, we reduced the level of EGFR mRNA using EGFR knockdown. The reduced expression level of EGFR mRNA was confirmed in EGFR siRNA‐treated ORS cells (Figure 6E). Interestingly, the expression level of EGFR mRNA was upregulated by EREG, suggesting that EGFR expression is positively regulated by EREG. EGFR knockdown eliminated stimulatory effect of EREG (Figure 6F,G). These results suggested that EREG binds to EGFR in ORS cells, thereby stimulating ORS cells.

As EREG is expressed in keratinocytes including epidermal and ORS cells in vivo (Figure 2), EREG mRNA expression is knockdown using EREG siRNA in vitro. The reduced expression level of EREG mRNA was confirmed in EREG siRNA‐treated ORS cells (Figure 6H). EREG knockdown decreased ORS cell proliferation and migration (Figure 6I,J). These results suggested that the released EREG from epidermal and ORS cells can stimulate ORS cells again as a positive feedback mechanism.

## 4 DISCUSSION

Whereas direct injection of HB‐EGF was not able to induce promotion of hair growth (Figure S1), direct injection of EREG significantly promoted hair growth (Figure 1). A recent study revealed that hEREG induced elongation of hair shaft stimulating keratinocytes.^12^ However, not much is known about the molecular mechanism of how EREG promotes hair growth. Our study examined how EREG can promote hair growth in vivo and ex vivo using human recombinant or knockdown of EREG. The present study also further investigated EREG expression in keratinocytes including epidermis and ORS cells, and differential activation and stimulation of EREG‐EGFR and EREG‐ErbB4 on ORS cells and DPCs, respectively. Moreover, the activation of EREG‐ErbB4 increases NOX4 expression, thus controlling the ROS level in DPCs.

There are controversial reports on the hair growth‐promoting effect of growth factors. For example, recombinant platelet‐derived growth factor (rhPDGF)‐A and rhPDGF‐B have been shown to induce anagen transition and increase the proliferation of DPCs. However, EGF mimetics inhibited hair growth in an animal model. Keratinocyte growth factor and EGF signalling inhibited hair follicle induction,^15^ and the EGFR decreased stathmin 1 and triggered catagen entry in the mouse.^16^ Our results were also controversial because direct injection of HB‐EGF did not affect hair growth promotion at all (Figure S1), whereas EREG directly induced hair growth promotion (Figure 1). These results were consistent with the report of another research group, which noted that PDGF‐AA‐inducible EREG promotes elongation of human hair shaft by enhancing proliferation and differentiation of follicular keratinocytes.^12^ This marks the first evidence that EREG has hair growth‐promoting effects directly. However, their study overlooked EREG‐receptor activation in DPCs and the role of DPCs as major storage to control the proliferation and differentiation of hair follicle stem cells and matrix cells. Our study further demonstrated that EREG phosphorylated ErbB4 on DPCs in vivo, and this EREG‐ErbB4 activation stimulated DPCs by regulating the ROS level through NOX4 expression. This represents the first evidence that one member of the EGF family stimulated DPCs via ErbB4 during hair cycling. In agreement with their report, our results also showed that EREG activated EGFR on keratinocytes including ORS cells in vivo. However, our results further revealed that a strong phosphorylated EGFR (p‐EGFR) expression occurred in matrix cells at anagen, suggesting that EREG‐EGFR activation may stimulate matrix cell proliferation for hair growth during the anagen stage. We may need to further see the relationship between MAPK/PI3K signalling and EREG‐EGFR activation in matrix cells, but we suspect that these signals may be involved in matrix cell proliferation. It has been reported that the EGF family triggered activation of the Ras‐Raf‐MAPK and PI3K pathways,^9^ which are representative signalling for cell proliferation.

It has been reported that EREG had a binding affinity for both EGFR and ErbB4.^10^, ^13^ The ErbB family comprises four homologous RTKs. The EGFR, ErbB3 and ErbB4 receptors can bind with ligands, whereas ErbB2 lacks a ligand binding domain and functions as a preferred co‐receptor. ErbB4 is the only ErbB family member that binds all four neuregulins, as well as EGF, EREG and HB‐EGF that had originally been identified as EGFR ligands.^13^ There are published reports about stimulation of DPCs by growth factor and receptor expression of some growth factors on DPCs. The intradermal adipocyte precursor cells activated PDGF‐PDGFR signalling in the DP in a dynamic manner.^17^ Our results also showed that EREG phosphorylated ErbB4 in DPCs and stimulated DPCs, as indicated by cell proliferation and the expression of DPC marker genes in vitro (Figure 4).

Hair follicle growth is dependent on the interaction between epithelial precursor cells that reside in the bulge region and DPCs.^18^, ^19^ DPCs provide the instructive signals required to induce the proliferation and initiation of anagen follicle growth of matrix cells as well as epithelial bulge stem cells.^20^, ^21^ Recent work has shown the identification of epithelial matrix progenitors within DPCs and its influence on the proliferative characteristic of the mature hair follicles, thereby indirectly regulating hair growth.^22^ Interestingly, although DPCs are responsible for controlling hair growth, it is not well understood how these cells are maintained or replenished, because cells within the DP rarely undergo mitotic division.^23^ Indeed, bromodeoxyuridine (BrdU) labelling or Ki67 staining trials revealed that proliferating DPCs were not detected in vivo. However, many previous studies have shown that DPC proliferation and the increased expression of DPC marker genes had a significant correlation relationship with hair growth promotion effect.^6^, ^24^ Our previous study also revealed that DPCs placed under hypoxic conditions showed increased proliferation and expression of DPC marker gene, and these DPC‐injected mice showed considerable hair growth in vivo.^14^

It has been reported that PDGF‐D mediated increased proliferation, migration and paracrine effects on ASCs via ROS generation.^3^ Although EGF mimetic reportedly affected the proliferation and differentiation of ASCs and mediated these functions via ROS generation in other cell type,^6^ this is the first evidence showing that EREG increases the mitogenic and hair growth‐promoting effects in DPCs via ROS generation. The ROS generating system was further examined, and EREG increased cellular ROS level, but did not lead to ROS generation in the mitochondria of DPC (Figure S7). The expression of Nox4 was high in the nucleus of DPCs,^14^ and Nox4 silencing attenuated the ROS level, NOX4 expression and DPC stimulation by EREG, suggesting that EREG increased ROS generation through nuclear NOX4 expression in DPCs. It was reported that amphiregulin (AREG) and HB‐EGF have the same effect at increasing NOX1 expression in smooth muscle cells.^25^ Although the scope of our study does not specifically cover more detailed transcriptional data, this marks the first evidence that EREG regulates NOX4 expression. Because EREG is a releasing growth factor, further research is warranted to determine which transcription factors are involved in NOX4 expression by EREG.

In summary, EREG induced hair growth as evidenced by recombinant and knockdown of EREG in vivo. EREG was expressed in keratinocytes including epidermal and ORS cells during hair cycling, and its expression level was high in anagen compared to telogen. Differential activation of EREG‐EGFR and EREG‐ErbB4 occurred on keratinocytes including matrix/ORS cells and DPCs, respectively, during hair cycling. EREG‐EGFR activation stimulated ORS cells as a positive feedback and EREG‐ErbB4 activation stimulated DPCs regulating ROS generation and NOX4 expression. Therefore, EREG therapy may offer a novel solution for hair loss treatment.

## CONFLICTS OF INTEREST

The authors declare no conflicts of interest.

## **AUTHOR** **CONTRIBUTIONS**

NC involved in conceptualization, data curation, formal analysis, investigation, methodology, validation, visualization and writing—original draft preparation. JS involved in conceptualization, funding acquisition, project administration, supervision, validation and writing—original draft preparation. WK involved in writing—review and editing. SO involved in writing—review and editing.

## Supporting information

Fig S1

Fig S2

Fig S3

Fig S4

Fig S5

Fig S6

Fig S7

Supplementary Material

## Data Availability

The data that support the findings of this study are available from the corresponding author upon reasonable request.
