# Supplementary figures and images for: Epiregulin promotes hair growth via EGFR‐medicated epidermal and ErbB4‐mediated dermal stimulation
Source: Cell Prolif. 2020 Jul 22;53(9):e12881. doi: 10.1111/cpr.12881 (PMC7503099; doi:10.1111/cpr.12881)

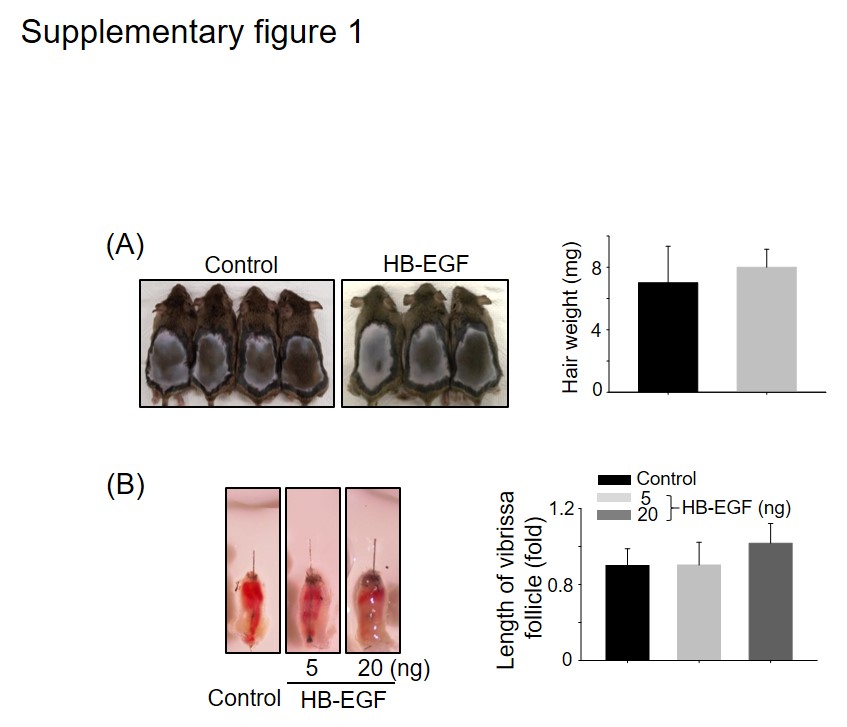

Supplement: Supplementary file 1 — Fig S1 [file CPR-53-e12881-s001.jpg]

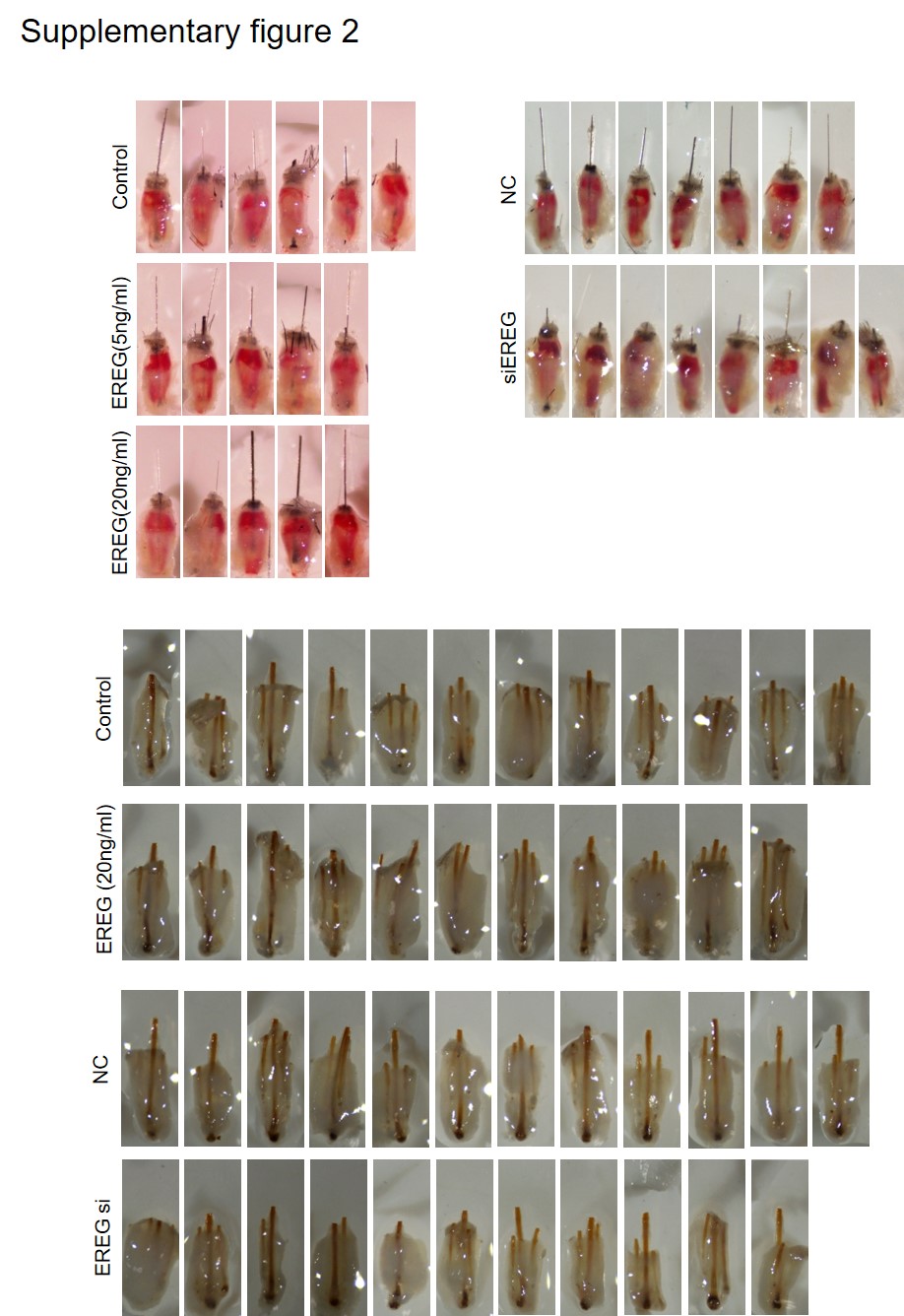

Supplement: Supplementary file 2 — Fig S2 [file CPR-53-e12881-s002.jpg]

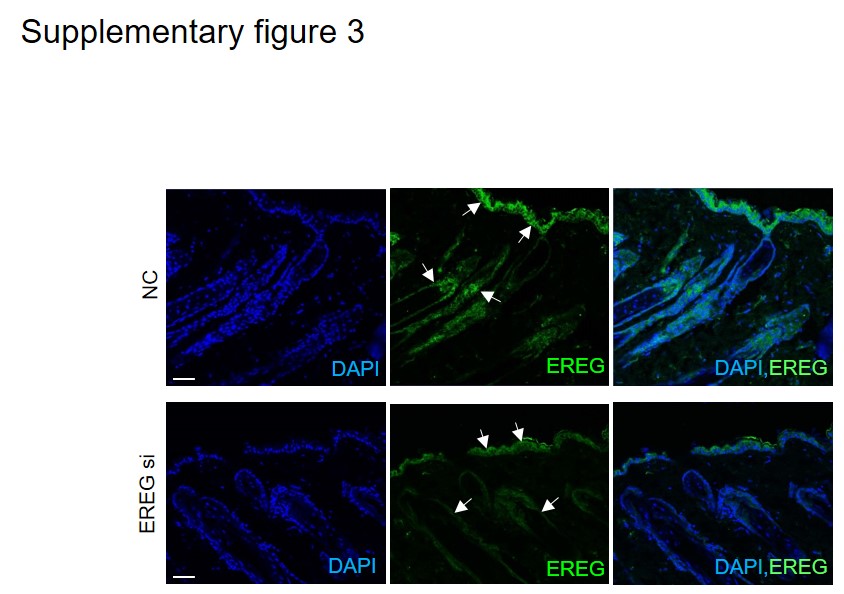

Supplement: Supplementary file 3 — Fig S3 [file CPR-53-e12881-s003.jpg]

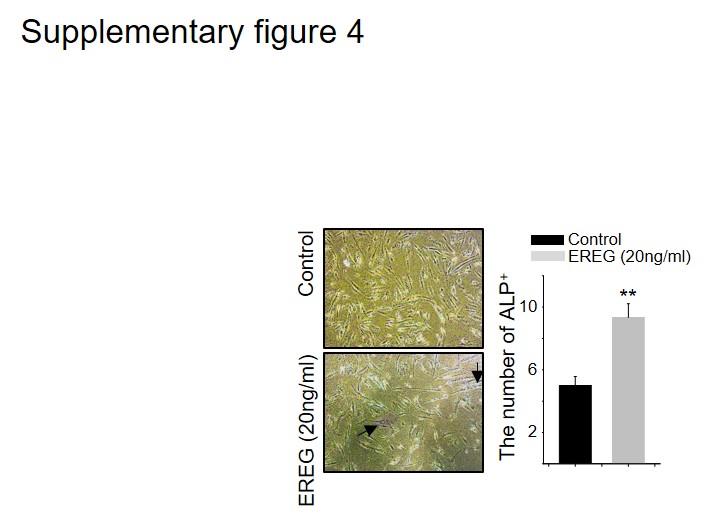

Supplement: Supplementary file 4 — Fig S4 [file CPR-53-e12881-s004.jpg]

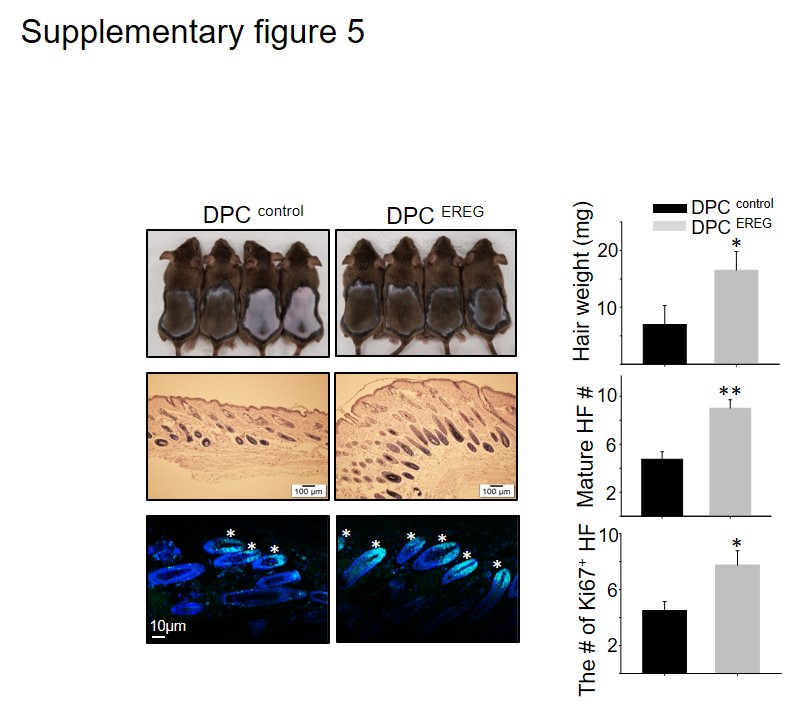

Supplement: Supplementary file 5 — Fig S5 [file CPR-53-e12881-s005.jpg]

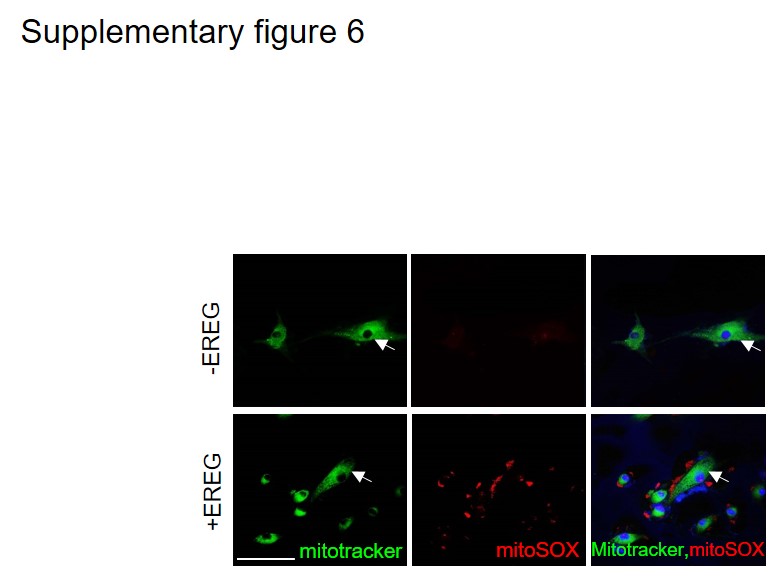

Supplement: Supplementary file 6 — Fig S6 [file CPR-53-e12881-s006.jpg]

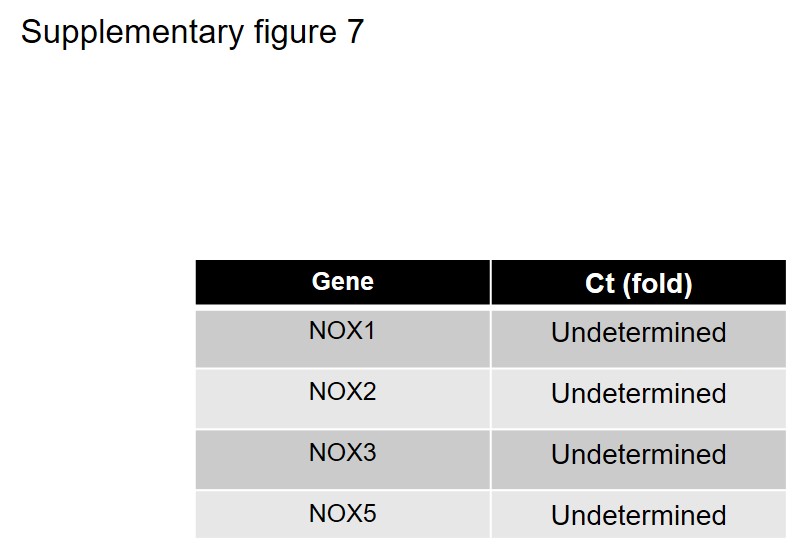

Supplement: Supplementary file 7 — Fig S7 [file CPR-53-e12881-s007.jpg]
