# Supplementary material for: Epiregulin promotes hair growth via EGFR‐medicated epidermal and ErbB4‐mediated dermal stimulation
Source: Cell Prolif. 2020 Jul 22;53(9):e12881. doi: 10.1111/cpr.12881 (PMC7503099; doi:10.1111/cpr.12881)
Supplement: Supplementary file 8 — Supplementary Material [file CPR-53-e12881-s008.docx]

Supplementary material

**Materials and Methods**

**Cell growth assay**

To measure cell growth, DPCs were seeded in 12-well plates at 5 × 10^3^ cells well^–1^, treated with EREG (5 or 20 ng/ml) 24 h later, and incubated for 4 days. Meanwhile, ORSs were seeded in collagen-coated 12-well plates at 1 × 10^4^ cells well^–1^, treated with EREG (5 or 20 ng/ml) 24 h later, and incubated for 5–6 days. To measure cell number, cells were then trypsinized (Gibco), stained with trypan blue (Sigma-Aldrich, St. Louis, MO, USA), and counted each day using a hemocytometer under a Nikon ECLIPSE Ts2 microscope.

**Cell migration measurement using transwell migration assay**

The ORSs were seeded into 60-mm plates (5 × 10^4^ per well), treated with EREG (5 or 20 ng/ml) 24 h later, and incubated for 3 days. The cells were then cultured in serum-free medium for 16 h, and then (2 × 10^4^ per transwell) seeded on the upper side of transwell membrane plates (BD Falcon, San Jose, CA, USA). Next, 700 μl medium containing serum was added to the lower chambers. The cultures were incubated for 24 h to allow transwell migration. To remove nonmigrated cells, the upper surface of the insert was cleaned with cotton swabs and washed with phosphate-buffered saline (PBS). The inserts were stained with 0.1% formalin/2% crystal violet solution (Sigma-Aldrich) for 30 min and dried. Multiple random images (10) per insert were captured using a ZEISS Axio Observer D1 microscope (Carl ZEISS, Jena, Germany), and the average number of cells were counted using Adobe Photoshop CS6 Extended program (Adobe Systems Inc., San Jose, CA, USA).

**NOX4 knockout**

For NOX4 knockout, DPCs were seeded in 60-mm dishes with approximately 50% confluence. The following day, 1μg of control or NOX4 CRISPR/Cas9 KO plasmid (Santa Cruz, California, USA) were transfected using Lipofectamin 2000 (Invitrogen, Waltham, Massachusetts, USA). Cells were incubated for 72 h after transfection, and puromycin (4μg/ml) was added to select drug-resistant pools at 48 h. NOX4 silencing DPCs were evaluated by using Western blotting^14^.

**ErbB4, EGFR and EREG knockdown**

For ErbB4 knockdown in DPCs, DPCs were seeded in 60-mm dishes with approximately 50% confluence. The following day, ErbB4 siRNA or negative control siRNA (bioneer, Daejeon, Korea) were transfected using Lipofectamin RNAi MAX (Invitrogen) and incubated for 3 days. ErbB4 knockdown in DPCs were evaluated by QPCR. For EGFR or EREG knockdown in ORS cells, same methods were used.

**Analysis of alkaline phosphatase activity**

DPCs were cultured in six-well plates with 70% confluency, treated with EREG (20 ng/ml) 24 h later, and incubated for 4–5 days. Then, the cells were fixed with 4% paraformaldehyde for 2 min, washed with PBS, stained with ALP staining solution (Alkaline Phosphatase Detection Kit CSR004; Millipore, Billerica, MA, USA) for 30 min, and then mounted with 80% glycerol.

**RNA extraction, quantitative RT-PCR, qPCR array, and RT-PCR**

Total RNA was extracted from the DPCs or ORSs using TRIzol reagent (Invitrogen, Grand Island, NY, USA). cDNA was synthesized using extracted RNA, oligodT, and the HelixCript™ Thermo Reverse Transcription system (NanoHelix, Madison, WI, USA) according to the manufacturer’s instructions. SYBRGreen qPCR master mix (Takara, Shiga, Japan) was used for quantitative polymerase chain reaction (qPCR) according to the manufacturer’s instructions. All primers are described in Supplementary Table S1.

**Hematoxylin and eosin staining**

For hematoxylin and eosin (HE) staining, paraffin sections were dewaxed three times using xylene for 15 min, and hydrated in 100%, 90%, 80%, and 70% ethyl alcohol (EtOH). Then, slides were dipped into Mayer’s hematoxylin (Sigma-Aldrich) for 10 min and rinsed in flowing water for 1 min. Next, slides were dipped into eosin Y (Sigma-Aldrich) for 1 min and 30 s and rinsed in flowing water for 1 min. Finally, slides were dehydrated with 70%, 80%, 90%, and 100% EtOH, washed twice with fresh xylene for 15 min, and then dried and mounted with mounting medium. Images were captured using a ZEISS Observer D1 microscope.

**Immunofluorescence staining**

The paraffin sections were dewaxed three times using xylene for 15 min and hydrated in 100%, 90%, 80%, and 70% EtOH. Antigen retrieval was performed using a microwave oven with boiling antigen retrieval solution (pH 6.0; Dako, Carpinteria, CA, USA) for 2 min 20 s. The sections were stained with rabbit Ki67 (1:300; Abcam, Iowa City, IA, USA), rabbit p-ErbB4 (1:300; Abcam), rabbit-p-EGFR (1:300; Abcam), EREG (1:300; Abcam), keratin17 (1:300; Santa Cruz Biotechnology, Dallas, TX, USA) antibodies overnight at 4°C, and then incubated with Alexa Fluor 488 goat anti-rabbit immunoglobulin G (IgG) (1:1,000; Invitrogen, Grand Island, NY, USA), and Alexa Fluor 594 goat anti-mouse IgG (1:1,000; Invitrogen, Grand Island, NY, USA) for 1 h at room temperature with 4,6-diamidino-2-phenylindole (DAPI; Sigma-Aldrich). For cell staining, the cells were fixed with 4% paraformaldehyde for 30 min at room temperature, washed with PBS, and incubated with p-EGFR1 and p-ErBb4 antibodies overnight at 4°C. The samples were then incubated with Alexa Fluor 488 goat anti-rabbit IgG (1:1000; Invitrogen, Grand Island, NY, USA) secondary antibodies for 1 h at room temperature with DAPI. Images of immunofluorescence staining were captured using a ZEISS LSM700 confocal microscope.

For immunostaining after EREG sc injection, back skin tissue was prepared at 15-20min after EREG (100 ng/ml) injection into the dorsal skin of mice. For immunostaining after EREG treat in cells, cells were seeded in 6-well plate with cover glass, treated with EREG (20 ng/ml) for 20 min and stained.

**Mouse vibrissae follicle and pig skin hair follicle organ culture**

For organ culture of the vibrissae follicles, vibrissae follicles were cut from C_3_H/HeN mice, washed with PBS, and then cultured in specified medium (Williams E medium supplemented with 2 mM l-glutamine, 10 µg/ml insulin, 10 ng/ml hydrocortisone, 100 U/ml penicillin, and 100 µg/ml streptomycin, without serum) with EREG (5, 20 ng/ml) for 2.5 days. For organ culture of the pig skin hair follicle, skin hair follicles were cut from a pig (Yorkshire–Landrace–Duroc hybrid), washed with medium (Williams E medium supplemented with 100 µg/ml streptomycin), and then cultured in specified medium (Williams E medium supplemented with 2 mM l-glutamine, 10 µg/ml insulin, 10 ng/ml hydrocortisone, 100 U/ml penicillin, and 100 µg/ml streptomycin, without serum) with EREG (20 ng/ml) for 5 days. Images were captured using an SMZ7457 microscope, and the average length of hair follicles was measured using Adobe Photoshop CS6 Extended program.

For siRNA treatment, mouse vibrissae and pig skin follicles were cut, washed, and cultured with EREG siRNA (Bioneer; 48 pmol/ 1 follicle), transfection reagent (*in vivo*-JetRNA), and 10% glucose with the above medium for 3 and 7 days, respectively.

**Statistical analysis**

All experiments in this study were performed more than three times with independent cultures. Data are presented as the mean ± standard error. The mean values were compared using Student’s *t*-test. For all statistical tests, a 0.05 level of confidence was considered statistically significant.

Supplementary table 1; Primers used for QPCR and PCR

| **Gene** | **Primer sequences (5’-3’)** |
| --- | --- |
| CD133 | AGTCGGAAACTGGCAGATAGC  GGTAGTGTTGTACTGGGCCAAT |
| ALP | GATCCTAAAAGGGCAGAAGA  GGCACATGCTTGTCTACACT |
| CORIN | CCAAAGCCGGTCTTGAGAG  GAGGAGGTTAGCAGTCGCC |
| NESTIN | TCTGGAAGAGGAAGACAACC  GTCCTTCTCCACCCGTATCTT |
| EGFR | AGGCACGAGTAACAAGCTCAC  ATGAGGACATAACCAGCCACC |
| ErbB4 | GTCCAGCCCAGCGATTCTC  AGAGCCACTAACACGTAGCCT |
| EREG | GTGATTCCATCATGTATCCCAGG  GCCATTCATGTCAGAGCTACACT |
| NOX4 | TGACGTTGCATGTTTCAGGAG  AGCTGGTTCGGTTAAGACTGAT |
| GAPDH | CGAGATCCCTCCAAAATCAA  TGTGGTCATGAGTCCTTCCA |
